# Supplementary material for: Vision-based detection and quantification of maternal sleeping position in the third trimester of pregnancy in the home setting–Building the dataset and model
Source: PLOS Digit Health. 2023 Oct 3;2(10):e0000353. doi: 10.1371/journal.pdig.0000353 (PMC10547173; doi:10.1371/journal.pdig.0000353)
Supplement: S1 Appendix — (PDF) [file pdig.0000353.s001.pdf]

S1 Appendix: Additional methodological details

Rationale for simulated posture study design

The data produced by an overnight (approximately eight hours) recording has a high probability of lacking heterogeneity because while pregnant people have been observed to change their sleeping position a median of six (IQR 3-10) times overnight during natural sleep in the third trimester,[1] they often only change between their favourite two or three positions, whereas our dataset needed rich representation of many different sleeping positions.

Bias-variance trade-off in k-fold cross-validation

There is a bias-variance trade-off associated with the choice of k in k-fold cross-validation; however, a k=5 or k=10 has been demonstrated to yield test error rate estimates that suffer neither from excessively high bias nor very high variance.[2]

Selection of YOLOv5s

Because of its speed and accuracy, You Only Look Once (YOLO) is one of the most famous algorithms used for object detection,[3,4] and the version YOLOv4 by Alexey Bochkovskiy is most well-known and cited.[5] In this study, we trained our model with an implementation of YOLO by Glenn Jocher called YOLOv5.[6,7] YOLOv5 uses the New CSP DarkNet53 as its backbone network, the SPPF and New CSP-PAN as its neck, and YOLOv3’s head as its head.[6] YOLOv5 has different models based on the number of parameters. We used YOLOv5s, which has a total of 7.5M parameters. The reason for choosing this model is because

of its performance on the standard COCO dataset and various other advantages including small size, speed, and implementation in the Pytorch framework.[6,8–10]

Early stopping criteria

In order to avoid overfitting, we used early stopping criteria. The early stopping patience was set to 20 epochs, that is, training of the model was stopped if the model did not show any improvement in training performance (validation mAP) in the previous 20 epochs. One epoch was the number of iterations it takes for the training process to “see” all the training frames in the training dataset. YOLO does not “see” all the training frames in each iteration; rather, in each iteration, YOLO only takes a “batch” from the total number of training frames, and we set our batch size as 16.

Definition of precision and recall

Precision for a given class is how likely the model prediction is true in the event that it predicts the given class. Recall for a given class is how well the model finds all the positives for that class (true positives), which is the same as “sensitivity” in the medical field. Note that “specificity” is not used in YOLO because YOLO is a detector and negative predictions are not generated (and thus true negatives cannot be derived, while false negatives can be derived allowing for calculation of recall or “sensitivity”).

References

1. Kember AJ, Scott HM, O’Brien LM, Borazjani A, Butler MB, Wells JH, et al. Modifying maternal sleep position in the third trimester of pregnancy with positional therapy: a randomised pilot trial. BMJ Open. 2018;8: e020256. doi:10.1136/bmjopen-2017-020256

2. James G, Witten D, Hastie T, Tibshirani R. Resampling Methods. An Introduction to Statistical Learning. Springer New York; 2013. pp. 175–201. Available: https://books.scholarsportal.info/en/read?id=/ ebooks/ebooks3/ springer/2013-08-13/1/9781461471387
3. YOLO: Real-Time Object Detection. [cited 7 Jun 2022]. Available: https://pjreddie.com/darknet/yolo/
4. Redmon J, Divvala S, Girshick R, Farhadi A. You Only Look Once: Unified, Real-Time Object Detection. arXiv; 2016. Available: http://arxiv.org/abs/1506.02640
5. Bochkovskiy A, Wang C-Y, Liao H-YM. YOLOv4: Optimal Speed and Accuracy of Object Detection. arXiv; 2020 Apr. Report No.: arXiv:2004.10934. doi:10.48550/arXiv.2004.10934
6. Home · ultralytics/yolov5 Wiki. In: GitHub [Internet]. [cited 7 Jun 2022]. Available: https://github.com/ultralytics/yolov5
7. YOLOv5 Documentation. [cited 7 Jun 2022]. Available: https://docs.ultralytics.com/
8. Jocher G, Chaurasia A, Stoken A, Borovec J, NanoCode012, Kwon Y, et al. ultralytics/yolov5: v6.1 - TensorRT, TensorFlow Edge TPU and OpenVINO Export and Inference. Zenodo; 2022. doi:10.5281/zenodo.6222936
9. Nepal U, Eslamiat H. Comparing YOLOv3, YOLOv4 and YOLOv5 for Autonomous Landing Spot Detection in Faulty UAVs. Sensors. 2022;22: 464. doi:10.3390/s22020464
10. Karthi M, Muthulakshmi V, Priscilla R, Praveen P, Vanisri K. Evolution of YOLO-V5 Algorithm for Object Detection: Automated Detection of Library Books and Performace validation of Dataset. 2021 International Conference on Innovative Computing, Intelligent Communication and Smart Electrical Systems (ICSES). 2021. pp. 1–6. doi:10.1109/ICSES52305.2021.9633834
