# Supplementary material for: Vision-based detection and quantification of maternal sleeping position in the third trimester of pregnancy in the home setting–Building the dataset and model
Source: PLOS Digit Health. 2023 Oct 3;2(10):e0000353. doi: 10.1371/journal.pdig.0000353 (PMC10547173; doi:10.1371/journal.pdig.0000353)
Supplement: S2 Appendix — (PDF) [file pdig.0000353.s002.pdf]

S2 Appendix: Collapsed-resolution model

Methods

We present the performance evaluation of the models from each loop under a collapsed resolution (CR) condition where we reduced the total number of classes by combining classes with similar anatomic and hemodynamic implications from the perspective of uteroplacental perfusion. In the CR models, we combined left recovery (P1), left lateral (P2), left tilt (P3), and supine thorax with left pelvic tilt (P7) into one class, “CR Left”. We combined supine (P4), supine pelvis with left thorax tilt (P5), and supine pelvis with right thorax tilt (P6) into one class, “CR Supine”, which requires a supine pelvis. We combined supine thorax with right pelvic tilt (P8) and right tilt (P9) into one class, “CR Right tilt”, because some studies have shown that right pelvic tilt may worsen IVC compression or fail to relieve it and result in reduced maternal cardiac output and stroke volume in comparison to the effect of left pelvic tilt.[1–7] Finally, we combined right lateral (P10) and right recovery (P11) into one class, “CR Right”. Sitting up at the edge of the bed (P12) remained as its own class, “CR Sitting”.

Results

S1 Fig shows a heatmap of precision, recall, AP@0.50, and AP@.50-.95 (columns) from the testing phase averaged across the six models’ test sets for each of the predicted collapsed-resolution (CR) classes (rows) under the “without bed sheets” and “with bed sheets” condition. The value of the respective performance parameter is mapped to a colour spectrum from red to yellow to green where values of 0.50 or less are represented by red at the lower end of the spectrum, values around 0.75 are shades around yellow (oranger if lower than 0.75; greener if

higher than 0.75), and values of 0.90 or more are represented by green at the higher end of the spectrum. The “all class average” is provided as the averaged value of the respective performance parameter across the six models’ test sets and the five CR classes under each bed sheets condition, and the “combined 24 class average” is given as the average of the former two values combined. For these “all class average” rows, the value in the AP@0.50 column is a mAP@0.50, and the value in the AP@.50-.95 column is a mAP@.50-.95 since these values represent averages across multiple classes.

On a per class basis under the “without bed sheets” condition, the AP@0.50 across the models was highest for the right CR class, followed by supine CR, left CR, and sitting, with the right tilt class having the lowest AP@0.50 across the models. When the “with bed sheets” condition is considered, the AP@0.50 across the models was highest for the sitting class, followed by left CR, right CR, and supine CR, with the right tilt class, as seen in the “without bed sheets” condition, having the lowest AP@0.50 across the models.

S1 Fig. **Heatmap of precision, recall, AP@0.50, and AP@.50-.95 (columns) from the testing phase averaged across the six models for each of the predicted collapsed-resolution (CR) classes (rows) under the “without bed sheets” (upper blue row header) and “with bed sheets” condition (lower yellow row header).** The value of the respective performance parameter is mapped to a colour spectrum from red to yellow to green where values of 0.50 or less are represented by red at the lower end of the spectrum, values around 0.75 are shades around yellow (oranger if lower than 0.75; greener if higher than 0.75), and values of 0.90 or more are represented by green at the higher end of the spectrum. The “all class average” is provided as the averaged value of the respective performance parameter across the six models’ test sets and the five collapsed-resolution classes under each bed sheets condition, and the combined “24 class average” is given (red column) as the average of the former two values combined. For these “all class average” rows, the value in the AP@0.50 column is a mAP@0.50, and the value in the AP@.50-.95 column is a mAP@.50-.95 since these values represent averages across multiple classes.

References

1. Fujita N, Higuchi H, Sakuma S, Takagi S, Latif MAHM, Ozaki M. Effect of Right-Lateral

Versus Left-Lateral Tilt Position on Compression of the Inferior Vena Cava in Pregnant Women Determined by Magnetic Resonance Imaging. Anesth Analg. 2019;128: 1217–1222. doi:10.1213/ANE.0000000000004166

2. Cluver C, Novikova N, Hofmeyr GJ, Hall DR. Maternal position during caesarean section for preventing maternal and neonatal complications. Cochrane Database Syst Rev. 2013; CD007623. doi:10.1002/14651858.CD007623.pub3
3. Fields JM, Catallo K, Au AK, Rotte M, Leventhal D, Weiner S, et al. Resuscitation of the pregnant patient: What is the effect of patient positioning on inferior vena cava diameter? Resuscitation. 2013;84: 304–308. doi:10.1016/j.resuscitation.2012.11.011
4. Bamber JH, Dresner M. Aortocaval Compression in Pregnancy: The Effect of Changing the Degree and Direction of Lateral Tilt on Maternal Cardiac Output. Anesth Analg. 2003;97: 256–258. doi:10.1213/01.ANE.0000067400.79654.30
5. Benninger B, Delamarter T. Anatomical factors causing oedema of the lower limb during pregnancy. Folia Morphol. 2013;72: 67–71. doi:10.5603/fm.2013.0011
6. Macklon NS, Greer IA, Bowman AW. An ultrasound study of gestational and postural changes in the deep venous system of the leg in pregnancy. BJOG Int J Obstet Gynaecol. 1997;104: 191–197. doi:10.1111/j.1471-0528.1997.tb11043.x
7. Humphries A, Thompson JMD, Stone P, Mirjalili SA. The effect of positioning on maternal anatomy and hemodynamics during late pregnancy. Clin Anat. 2020;33: 943–949. doi:10.1002/ca.23614
