# Supplementary figures and images for: Vision-based detection and quantification of maternal sleeping position in the third trimester of pregnancy in the home setting–Building the dataset and model
Source: PLOS Digit Health. 2023 Oct 3;2(10):e0000353. doi: 10.1371/journal.pdig.0000353 (PMC10547173; doi:10.1371/journal.pdig.0000353)

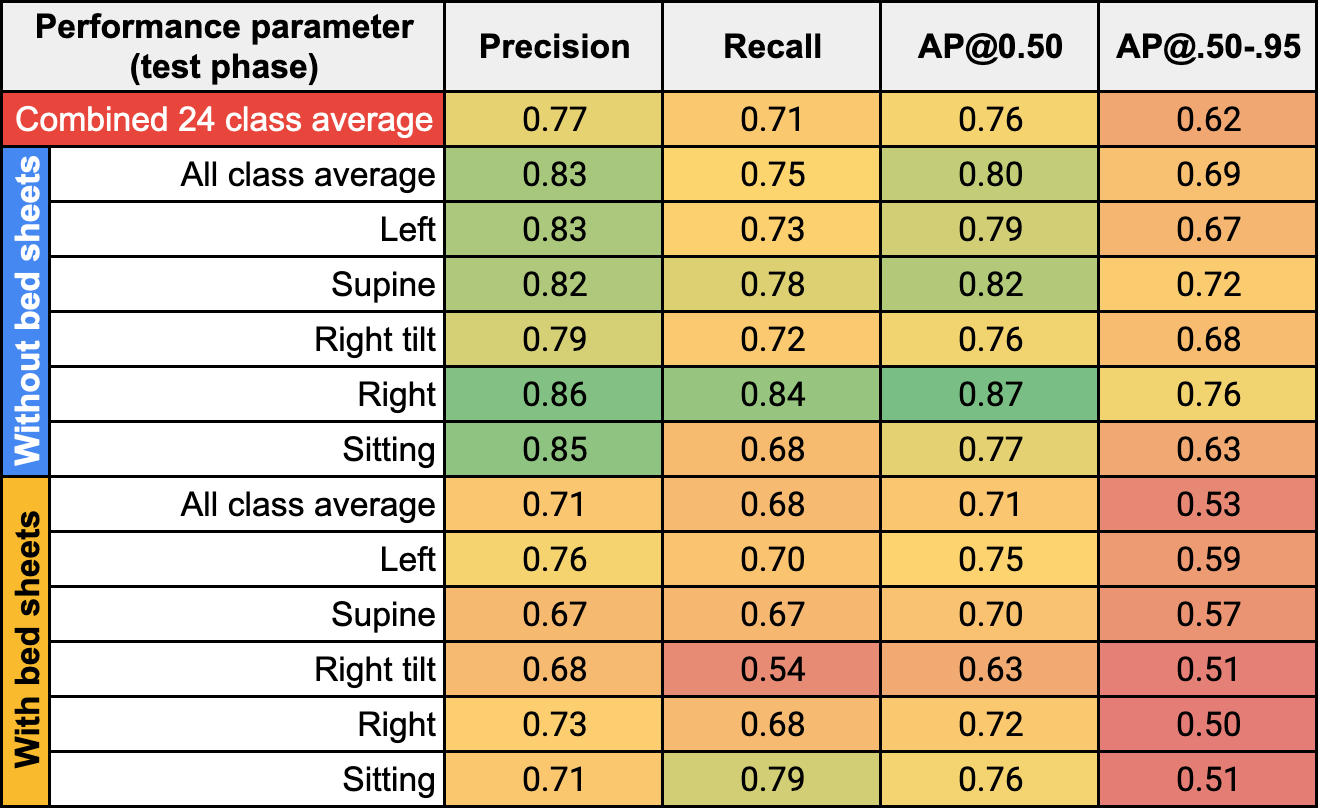

Supplement: S1 Fig — The value of the respective performance parameter is mapped to a colour spectrum from red to yellow to green where values of 0.50 or less are represented by red at the lower end of the spectrum, values around 0.75 are shades around yellow (oranger if lower than 0.75; greener if higher than 0.75), and values of 0.90 or more are represented by green at the higher end of the spectrum. The “all class average” is provided as the averaged value of the respective performance parameter across the six models’ test sets and the five collapsed-resolution classes under each bed sheets condition, and the combined “24 class average” is given (red column) as the average of the former two values combined. For these “all class average” rows, the value in the AP@0.50 column is a mAP@0.50, and the value in the AP@.50-.95 column is a mAP@.50-.95 since these values represent averages across multiple classes. (TIF) [file pdig.0000353.s003.tif]
