# Supplementary material for: Vision-based detection and quantification of maternal sleeping position in the third trimester of pregnancy in the home setting–Building the dataset and model
Source: PLOS Digit Health. 2023 Oct 3;2(10):e0000353. doi: 10.1371/journal.pdig.0000353 (PMC10547173; doi:10.1371/journal.pdig.0000353)
Supplement: Attachment — Submitted filename: Response to Reviewers.docx [file pdig.0000353.s004.docx]

Reviewer 1

| **Reviewer Comment** | **Our Response** |
| --- | --- |
| This study reports on an attempt to assess body position during sleep using video imaging and computer technology. The goal is to fill the gap in this technology by including pregnant people in order to better assess sleep position and pregnancy prospectively. The need to fill this gap is adequately established and the modeling is detailed and extensive. | Thank you. |
| My primary concern with the study is the possibility of generalization to real world views. The fact that they required no additional pillows in the sleeping area set up an unrealistic sleep environment for the pregnant person. In the third trimester, the majority of pregnant people support their bodies in various positions using a combination of traditional pillows, body pillows, and sleep wedges. These additions may make this technology no longer functional, and I would have liked to see at least some attempts at incorporating these sleep aids in the videos. | Thank you for noting this concern. It is a valid concern, and we hope that we have addressed it fully here and in our responses to your additional elaboration regarding this concern in the rows below.  First, we would like to point out that real-world generalizability of the present model is not our main objective in this study. In machine learning and software applications, it is standard practice to continually iterate and update one’s model by adding new data to the dataset and re-training, re-validating, and re-testing it (see, for example, Grandlund et al. 2021 [1]) Therefore, we have noted in our Discussion that the present model is not ready for real-world use and that we are currently collecting real-world data on which the present model will be trained further, which will increase generalizability for real-world use.  Second, we contend that, specifically for a simulation study as we conducted here, exclusion of pillows (except for head pillows) from the sleeping area will have a lesser impact on generalizability than one may think. This is because in a simulation study, participants were asked and coached to adopt specific body positions in real-time by the researcher who was watching the live video feed remotely. We fully agree that if this was a study to collect real-world video data of natural overnight sleep, restricting pillow use to the exclusion of all pillows except for head pillows would greatly impact real-world generalizability because it is likely that these other pillows and sleeping aids would have an effect on the sleeping positions encountered in the collected dataset.  Third, the present model is not trained to detect pillows. It is trained to detect body position regardless of pillows or other objects obscuring the body such as bedsheets. In a model that is trained to detect pillows in addition to body position, i.e., a model that is ready to be used in the real world, we agree with the reviewer that requiring no additional pillows would greatly impact real-world generalizability.  Please see our responses below as several changes were made throughout the manuscript to address your main primary here. |
| Author summary, line 46: use the phrase “sleeping on the back” but the more common term used in the literature is “supine sleep”. You could consider replacing this for consistency. | Thanks for this suggestion; however, from PLOS, “*The aim of the Author Summary is to make your findings accessible to a wide audience that includes both scientists and non-scientists*.” The reason that we used “sleeping on the back” rather than “supine sleep” is because a non-scientist audience is not likely to understand the meaning of “supine”. For example, the corresponding author (AJK) did not learn about the term “supine” until he was in medical school. |
| Introduction, line 76–77: there are other risk factors with far larger effect sizes and then many of these, such as pregestational diabetes, lupus, and previous pregnancy morbidity. Consider limiting this to a list of potentially modifiable factors. I appreciate, however, that you acknowledge that weight and body size is not realistically modifiable for the majority of people. | Thanks. Agreed. We have updated the manuscript to indicate that this list is a list of potentially modifiable risk factors. |
| Introduction, lines 81–88: this paragraph could be moved to the discussion. | Noted. |
| Introduction, lines 1 27–1 43: the majority of this could also be moved to discussion. | Noted. |
| Materials and methods, line 194–195: this is incorrect. You did, in fact, collect personal health information as names and addresses are part of personal health information. | Thanks. Agreed. We updated this to indicate that no additional personal health information (besides name, mailing address, age, gestational age, height, and weight) was accessed or shared. |
| Materials and methods, lines 211–212: this is my main challenge and accepting the importance of this paper. Removal of all pillows and objects from the bed except for head pillows is not consistent with how the majority of their trimester pregnant people sleep. Please either include an arm in which people do have pillows and wedges or justify the exclusion of this. | We acknowledge this concern. To address it, we have gone through our manuscript to dial down the enthusiasm of our manuscript vis-à-vis our model being ready for use as a tool by researchers in the real world. We have also emphasized this as a limitation and introduced the limitations paragraphs in the Discussion with, “*Our study has two main limitations that prevent our model from being ready for deployment in the real-world,*” to make it clear to the readership that, as is, while our model can account for some conditions of the natural sleeping environment (i.e., twisted and hybrid body positions, dark or low lighting, presence of thin pattern-free bedsheets), it cannot account for several other common conditions (i.e., bed partner, additional pillows besides head pillows, thick bedsheets, patterned bedsheets).  We edited the manuscript throughout to indicate that while our aim in the present paper is to see if we could build a dataset and model for sleeping position detection in pregnancy in the home setting, we do this with an eventual goal of our model to be the foundation of tool that researchers in the real-world can use. We feel that this eventual goal is a necessary piece of context to keep in the paper and have kept it in the Abstract and Discussion; however, we completely removed it from the Introduction section to emphasise to the reader that what we are building is not yet ready for real-world deployment. Can we account for many different body positions (other than left, right, supine, prone) like twisted and hybrid positions? How does the presence or absence of bedsheets affect accuracy? And can we account for dark and low-light conditions? These are the basic questions that we sought to explore in building the model in our current paper.  The word “tool” carries the connotation of real-world use; therefore, we also edited the manuscript throughout and made a deliberate effort to use the word “model” to describe what we have built in this study and reserve the term “tool” for the eventual goal of our model or to refer to devices that are already being used or should be used in the real-world. |
| Materials and methods, line 306: this is a typo; the IVC runs up the right side of the spinal column. | Thanks for catching this. Corrected. |
| Results, table one: this is a fairly narrow and low range of BMIs. A limitation of your study is also generalizability to higher BMI, which is common in pregnancy and is a specific concern with regards to sleep position. | Thanks. We agree and have added this as a limitation in the Discussion as, “*The range of our participants’ BMI’s was relatively narrow and clustered in the normal and overweight category, which limits generalizability to pregnancies with higher BMI categories (e.g., class II obesity), which are common and a specific concern vis-à-vis sleeping position.*” |
| Discussion, lines 494–495: this is conjecture and not clear from what you present. | Agree. We have deleted this statement from the manuscript. |
| Discussion, lines 538–545: I would emphasize these limitations more strongly and included acknowledgment that many pregnant people sleep in a bed full of support pillows. Also acknowledge limited BMI range. | Thanks. We concur and have updated this section to emphasize that these are major limitations especially in light of eventual real-world deployment (as above). We also added that pregnant people commonly sleep with multiple pillows to support their body, which our current model does not account for. We also acknowledged that generalizability is impacted by our inclusion of a limited range of BMI’s (as above). |

Reviewer 2

| **Reviewer Comment** | **Our Response** |
| --- | --- |
| This article presents an automated analysis technique to identify sleep position in pregnancy. This is a worthwhile tool to develop, due to the lack of data on changes in sleep position in late pregnancy, where supine sleep is a modifiable risk factor for stillbirth. Participants were videoed sleeping in their own homes, but on beds with light unpatterned sheets (or no sheets) and with no pillows interfering with the field of view. | Thank you for this summary. |
| The motivation methods and results are well written and clear. However, given the results are limited by the set up of the participant imaging I would expect more detailed comment on the possible loss ability of the method to detect sleeping positions with these simplifying factors removed.  Patterned sheets or a co-sleeper would reduce accuracy, are there possible (more advanced?) techniques that could be tried in the future to remedy this? | Thanks. We agree with this assessment of this limitation. As noted in the last sentence of the second-last paragraph of our Discussion, we are currently addressing this limitation by collecting a real-world dataset, which has the presence of a co-sleeper, patterned and stripped bedsheets, thick bedsheets (e.g., Duvets), and multiple pillows (e.g., pregnancy pillows and wedges). In the Discussion, we have added an additional comment that accuracy would likely deteriorate if these simplifying factors were removed.  To answer your question regarding techniques: On our real-world dataset, we’ve used the same model building methodology as used in this manuscript (training, validation, and testing by six-fold cross validation using a deep learning neural network) and the results are promising but outside of the scope of the present manuscript and not yet ready to be published. |
| Related to this is the need for a clearer discussion on what kind of accuracy might be acceptable for the algorithms to meet their purpose in clinical and research studies. It is clear that the accuracy in your method is comparable to other methods for detecting sleep position, but what would be an acceptable accuracy for this type of study for this type of algorithm to replace, say, manual identification of sleep position? How close is your algorithm to this desired accuracy? | Thanks for this excellent and practical question. We have added more information in the Discussion to this regard, specifically, while the American Academy of Sleep Medicine's Manual for the Scoring of Sleep and Associated Events states that body position reporting is required polysomnographic studies and optional for home-based studies, there is currently no consensus on how accurate body position reporting should be.[2] Furthermore, standard research and clinical scoring of body position is limited to left, right, supine, prone, or upright;[2] however, for physiologic reasons unique to pregnancy, our models score body position at a higher resolution by accounting for body twist and hybrid positions (e.g., supine thorax with left pelvic tilt). As such, even if there was consensus on how accurate body position reporting should be, this would need to be extrapolated as eight of our models’ eleven detected sleeping positions are non-standard positions.  In summary, because there is no consensus on how accurate body position scoring should be, we feel that it is premature to discuss this matter further beyond what we have now added to the Discussion. |
| The article refers to a website that will in the future allow researchers to conduct similar analyses. When clicking on the weblink the website states “Coming august 2023” – if the website is not currently available, it can’t be reviewed and I question whether the link should be provided. | Noted. We have removed the link and all references to making our current tool, as presented in this manuscript, available to other researchers. We admit that our original manuscript was over-enthusiastic about our tool’s preparedness for use in the real-world, especially given the limitations that you and the other reviewer pointed out (i.e., no pillows, no bed partner, no thick bedsheets, no patterned bedsheets). The weblink was contributing to this over-enthusiasm, so we have removed it from the manuscript. |

**References**:

1.	Granlund T, Stirbu V, Mikkonen T. Towards Regulatory-Compliant MLOps: Oravizio’s Journey from a Machine Learning Experiment to a Deployed Certified Medical Product. SN Comput Sci. 2021;2: 342. doi:10.1007/s42979-021-00726-1

2.	The AASM Manual for the Scoring of Sleep and Associated Events. Version 2.6. American Academy of Sleep Medicine; 2020.
